# Identification of A Novel Picorna-Like Virus, Burpengary Virus, that is Negatively Associated with Chlamydial Disease in the Koala

**DOI:** 10.3390/v11030211

**Published:** 2019-03-02

**Authors:** Erin Harvey, Danielle Madden, Adam Polkinghorne, Edward C. Holmes

**Affiliations:** 1 Marie Bashir Institute for Infectious Diseases and Biosecurity, Charles Perkins Centre, School of Life and Environmental Sciences and Sydney Medical School, The University of Sydney, Sydney, NSW 2006, Australia; erin.harvey@sydney.edu.au; 2 Animal Research Centre, University of the Sunshine Coast, Sippy Downs, QLD 4556, Australia; danielle.madden@research.usc.edu.au (D.M.); apolking@usc.edu.au (A.P.)

**Keywords:** virus discovery, *Chlamydia pecorum*, koala, picornavirus, phylogeny

## Abstract

Koalas (*Phascolarctos cinereus*) are native Australian marsupials whose populations are in decline from a range of threats. Infectious diseases caused by the bacterium *Chlamydia pecorum* and other pathogens are of particular concern. We analysed 26 poly-A selected RNA-sequencing libraries from a data set designed to study the immune response of koalas to ocular chlamydial infection. Using virus discovery techniques, we identified the coding-complete genome sequence of a novel picorna-like virus, denoted Burpengary virus, that was most common in south-east Queensland. Notably, abundance measurements of the virus across all 26 libraries revealed an inverse relationship between abundance and ocular disease in koalas, suggesting that the co-infection of Burpengary virus and *Chlamydia pecorum* is inhibited.

## 1. Introduction

The koala (*Phascolarctos cinereus*) is an iconic Australian marsupial species under threat from a number of anthropogenic factors such as loss of habitat due to deforestation and urbanisation, attack from domestic animals such as cats and dogs, and vehicle collisions [1]. It has been estimated that the koala population of south-east Queensland has declined by as much as 80% since the 1990s. Shrinking population sizes and habitat regions are also contributing to the spread of disease. The infectious threat of most concern to the koala is *Chlamydia pecorum*, a bacterium that infects the eyes and urinary tract causing blindness, infertility and death [2]. It has been estimated that up to 100% of some koala populations are now infected with the bacterium, with most wild populations across Australia affected and those from northern Queensland experiencing higher levels of both infection and clinical disease [3,4].

In addition to *Chlamydia*, koalas are also known to be susceptible to a number of viral infections such as koala retrovirus (KoRV) and two gammaherpes viruses [5,6]. KoRV has been linked to leukaemia in koalas and is a potential contributing factor to the high rates of *Chlamydia*, such that KoRV might cause immunomodulation and increase susceptibility to chlamydial disease [7]. To determine if koalas suffering ocular disease due to *Chlamydia* might be infected with other viruses, we employed virus discovery techniques based on bulk RNA sequencing [8,9,10]. These data were previously collected to analyse the immune response of koalas to ocular infection, particularly that caused by *C. pecorum* [11].

## 2. Materials and Methods

### 2.1. Analysis of RNA Libraries and Identification of A Novel Virus

A total of 26 previously determined poly-A selected RNA-seq libraries of ocular tissue sampled from koalas with evidence of gross pathology consistent with koala chlamydiosis (*n* = 13), and those without (*n* = 13), were assembled and analysed [11]. All sequencing data is available at the NCBI SRA under Bioproject PRJEB26467. To identify novel viruses within the data set, the paired end reads were trimmed using Trimmomatic (v 0.36) [12] and assembled *de novo* using Trinity (v.2.5.1) [13]. A blast search of the assembled contigs was performed against the NCBI nr database using diamond blast (v 0.9.10) [14]. Hits to virus associated proteins were extracted and a subsequent blast analysis was run against the NCBI nt database using BLASTn [15] to determine if any of these contigs showed significant sequence similarity to previously characterised non-viral sequences. Contigs showing sequence similarity to viral proteins exclusively were then checked for complete open reading frames (ORFs) using the ExPASy translate tool (https://web.expasy.org/translate/). The coding-complete genome sequence of a picorna-like virus was identified in the assembled contigs of four libraries, while partial sequences were identified in nine other libraries. Bowtie2 (v.2.2.5) [16] was then used to retrieve full genomes from all other libraries, where possible using the Trinity contig as a reference. The genome structure of the novel virus was predicted based on the single ORF predicted, and a web blast conserved domain search [17] against the conserved domain database (CDD) was used to predict conserved protein regions.

### 2.2. Evolutionary Relationships and Viral Abundance

To determine the phylogenetic relationship of the novel virus identified here to previously characterised viruses the amino acid sequence of the assembled polyprotein was aligned to other viruses within the family *Picornaviridae*. Accordingly, all *Picornaviridae* polyprotein amino acid sequences available within the RefSeq database as of January 2019 were compiled and aligned to the novel protein sequence using MAFFT (v.7.300) [18] employing the L-INS-I algorithm. TrimAL (v.1.4.1) [19] was then used to remove ambiguously aligned regions. The IQ-tree (v.1.6.1) tool, ModelFinder [20], was used to determine the best-fit model of amino acid substitution - identified as the Le-Gascuel (LG) model. A maximum likelihood tree assuming this model was then inferred using PhyML (v.20150415) [21], with 1000 bootstrap replications used to determine the support for each node within the tree.

The abundance of the virus in each library was determined using Bowtie2 (v.2.2.5) [16] to align the fastq reads to the assembled genomes of the novel picorna-like virus. The abundance of the housekeeping gene GAPDH and KoRV were also measured using the same method with reference sequences of koala GAPDH and KoRV taken from the RefSeq database. The Bowtie2 read counts were converted to reads per kilobase million (RPKM) to normalise across the three references.

### 2.3. C. pecorum Abundance

*C. pecorum* infectious load was assessed in conjunctival swabs from each animal following DNA extraction as previously described [22]. A recently described *C. pecorum* species-specific LAMP assay was modified for qPCR analysis [23]. The F3 forward primer (5′ ATCGGGACCTTCTCATCG 3′) and B3 reverse primer (5′ GCTGTTGTAAGGAAGACTCC 3′) amplify a 209 base pair target region of the gene specific to *C. pecorum.* All reactions were run in duplicate and were carried out on a Rotor Gene Q real-time PCR machine in a 20 µL reaction volume. The reaction mix consisted of 10 µL QuantiTect mastermix containing SYBR Green I chemiluminescent dye, 1 µL of 10 µM each forward and reverse primer, 3 µL dH_2_O and 5 µL sample DNA template. Cycling conditions included an initial denaturation of 94 °C for 10 min, with 35 cycles of denaturation (94 °C for 15 s), annealing (57 °C for 30 s) and extension (72 °C for 25 s). Negative (dH_2_O) and positive (cultured *C. pecorum* DNA) controls were included in each run. Infection loads were expressed as a number of gene target copies/µL.

## 3. Results

### 3.1. Data Selection

A total of 26 sequencing libraries were chosen from a larger data set originally generated to analyse the immune response of koalas to ocular chlamydial infection [11] (those libraries excluded from our analysis had incomplete information on *C. pecorum* status and geographic location). These RNA-sequencing libraries included samples from 26 individual koalas in varying stages of ocular health grouped into five categories based on disease state and presence or absence of *C. pecorum* following qPCR screening. Specifically, (i) H1 indicates individuals with no signs of ocular disease and the absence of *C. pecorum*; (ii) H2 indicates individuals with no signs of disease but with *C. pecorum* detected by PCR; (iii) G1 are individuals with acute active disease but an absence of symptoms of chronic ocular disease; (iv) G2 indicates individuals with chronic, active disease; and (v) G3 are individuals with chronic, inactive disease with little or no discharge but evidence of corneal scarring.

### 3.2. Read Assembly and Analysis

Between 62,555,754 and 132,486,302 paired end reads were generated for each library, which we assembled into between 55,840 and 174,502 contigs using a *de novo* transcript assembly tool. These contigs were submitted to a series of blast searches to exclude host reads and determine if any viruses were present. As expected, KoRV was found in all 26 libraries, although it is challenging to distinguish exogenous from endogenous copies on these data. No other previously identified viruses were found.

### 3.3. Virus Discovery and Characterisation

**Figure 1:**
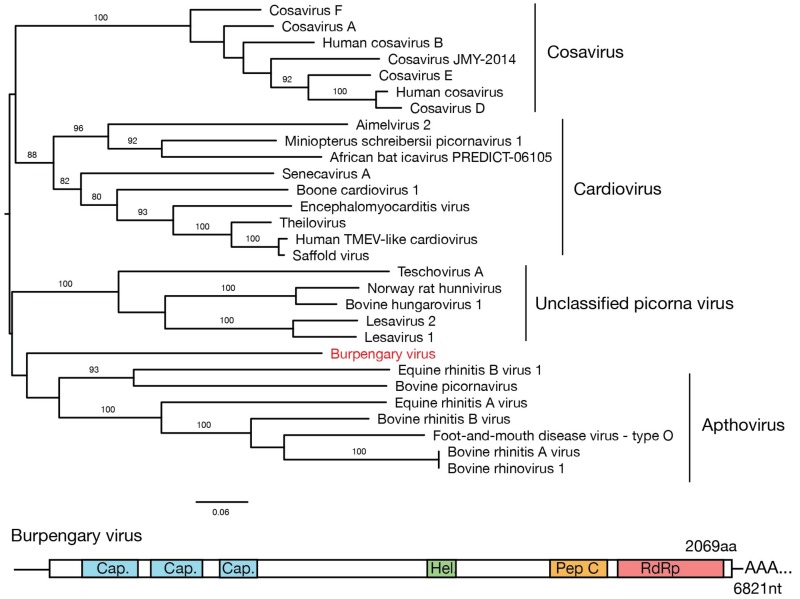
Maximum likelihood phylogenetic tree showing the position of the newly identified Burpengary virus (in red) in relation to other members of the *Picornaviridae*. The tree is midpoint rooted for clarity, and bootstrap support values of over 70% are shown. Genera within the *Picornaviridae* are indicated to the right of the tree. The predicted genome structure of the novel virus is shown below the phylogeny, with the single coding-complete ORF and conserved regions indicated as: Cap—the conserved picornavirus capsid protein domain, Hel—the conserved RNA helicase domain, Pep C—the peptidase C3 cysteine protease domain, and RdRp—the conserved RNA-dependent RNA polymerase.

A single novel positive-sense single-stranded RNA virus, which we refer to as Burpengary virus (named after the locality where it was found at highest prevalence), was identified in 15 libraries and a coding complete genome was assembled. A single ORF was predicted, suggesting a polyprotein organisation, with is consistent with the genome structure of other viruses within the family *Picornaviridae*. Within the predicted polyprotein a number of conserved protein sequences were identified using a blast conserved domain search consistent with the polyprotein structure of other viruses within the family (Figure 1). Analysis of the predicted coding-complete polyprotein amino acid sequence revealed that Burpengary virus is phylogenetically distinct, but most closely related to members of the *Apthovirus* genus that contains Foot-and-mouth virus, Equine rhinitis virus, Bovine picornavirus and Bovine rhinovirus, although with a marked lack of bootstrap support (Figure 1).

### 3.4. Abundance Measurement

**Figure 2:**
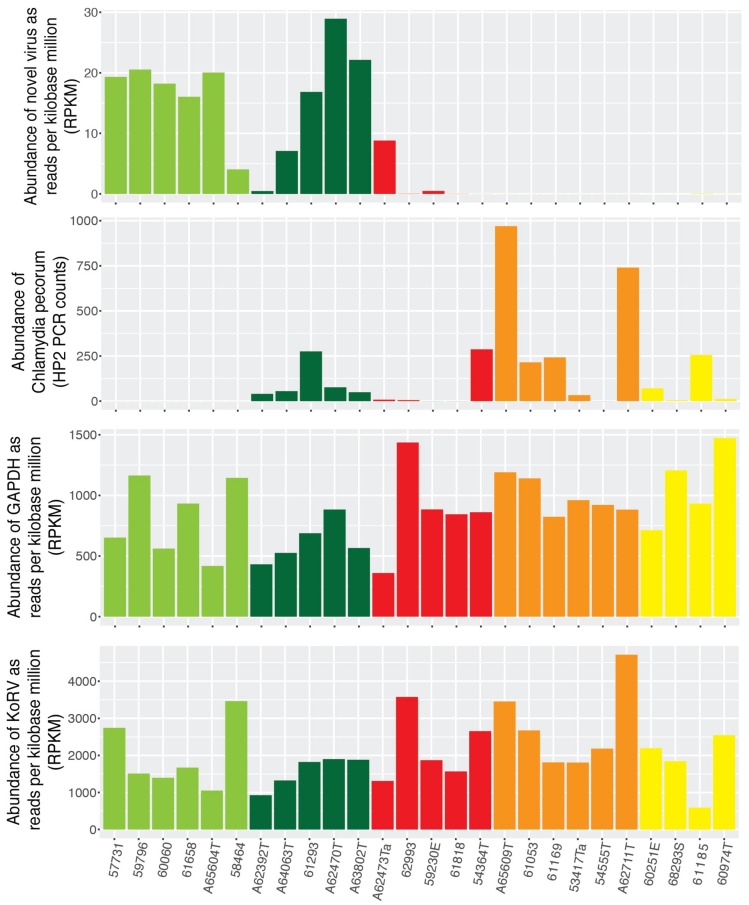
Abundance of Burpengary virus, koala GAPDH, KoRV and *C. pecorum*. Samples are grouped and colour coded by the individual koalas health status as follows: H1—light green, H2—dark green, G1—red, G2—orange and G3—yellow. The abundance of Burpengary virus, KoRV and GAPDH are measured in reads per kilobase million based on fastq reads, while the abundance of *C. pecorum* was measured by PCR.

The abundance of Burpengary virus was measured as reads per kilobase million, and compared to that of koala GAPDH and KoRV to determine if virus abundance was associated with the general abundance of transcripts within each data set. This revealed no association between the abundance of GAPDH or KoRV and Burpengary virus (Figure 2). Importantly, however, when samples were separated by disease status, the presence of Burpengary virus appeared to show an inverse relationship to signs of ocular disease, being highest in the H1 and H2 samples, and lowest in G1–G3 that designate acute or chronic *C. pecorum* infection (Figure 2). Due to the poly-A selection step being included in the library preparation of these samples, it is impossible to directly compare the abundance of *C. pecorum* with the virus and host genes. The abundance of *C. pecorum* was, however, measured using PCR and gene copies per µL of DNA extract from each conjunctival were recorded for each sample (Figure 2).

### 3.5. Geographical Distrivution of Burpengary Virus

**Figure 3:**
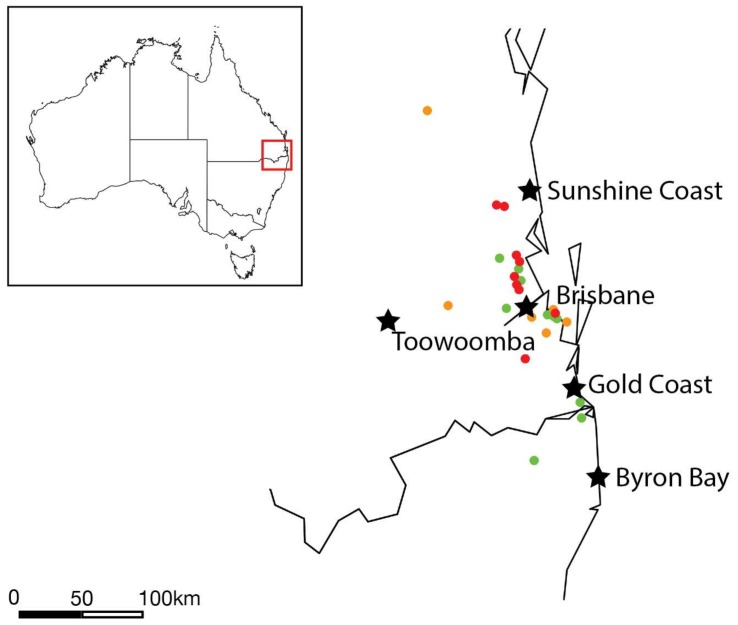
Map showing the koala collection area spanning from north of the Sunshine Coast, Australia, across the New South Wales and Queensland boarder to Byron Bay in the south. Key urban areas are indicated by black stars, while the koala collection locations are colour coded by virus abundance with green points indicating no virus with the sample, yellow indicating low levels of virus (below 0.01% of total reads) and red indicating high abundance of virus (>0.01% of total reads).

Koala samples were collected across south-east Queensland and north-east New South Wales, Australia. To tentatively determine if Burpengary virus was associated with a particular geographical region, the collection locations were mapped and collection points were coloured by virus abundance (Figure 3). This revealed that koalas infected with Burpengary virus were located further north in the collection area, with no virus seen in any samples collected from the border or NSW region. However, this may be an artefact of the distribution of collection locations as most samples were collected around the broader region of south-east Queensland and may be impacted by animal displacement as the koalas tested mainly come from care centres.

## 4. Discussion

Infectious diseases are a significant driver of morbidity and mortality in wild koalas [1]. The data set analysed here was designed to study the immune response of koalas to ocular chlamydial infection, and we subsequently analysed 26 poly-A selected sequencing libraries to identify any novel viruses. The technique of eliminating host reads and performing blast searches against a data set of conserved virus protein sequences such as the RNA-dependent RNA polymerase region of RNA viruses has been used previously to identify novel viruses [9,10,24,25]. We implemented this data analysis methodology here and in doing so identified a novel and phylogenetically distinct picornavirus that we have termed Burpengary virus. 

As the sequencing libraries were poly-A selected during library preparation, bacterial sequences are necessarily eliminated, such that we were unable to measure the abundance of *C. pecorum* relative to the abundance of Burpengary virus, KoRV or the housekeeping gene GAPDH [11]. This also limits the discovery of novel viruses within the samples as all non-polyadenylated viruses would be excluded from sequencing, and may be a major reason why only a single novel virus was identified. However, Burpengary virus was found in a large proportion of the sequenced samples, with 15 of the 26 libraries containing virus reads. It should be noted that due to the nature of the available data, the KoRV subtypes were not identified within this study. As insertions can be used to differentiate the subtypes by size [26], the most effective method of determining KoRV subtypes is by PCR targeting of the variable region of the virus. The expression levels of KoRV observed here are consistent with those observed in previous transcriptomic studies, and aside from outliers are similar to the expression of GAPDH [27].

Interestingly, Burpengary virus was identified almost exclusively in samples collected from healthy koalas showing no signs of ocular chlamydiosis. This tentatively suggests that there is a mechanism, currently unknown, that may prevent the co-infection of ocular tissues with the novel virus and *C. pecorum* when animals develop acute or chronic ocular disease, although this clearly needs verifying with a larger sample size. It is possible that infection with the bacterium may block viral replication in a similar manner to that of *Wolbachia*—a genus of arthropod-associated intracellular bacteria known to block the replication of a variety of RNA viruses in *Drosophila* and some mosquito species [28,29], including Dengue virus in *Aedes aegypti* mosquitoes [30,31]. A perhaps more likely possibility is that chlamydial infection of the mucosal surface by this intracellular bacterial pathogen promotes a strong local immune response, critical to chlamydial infection clearance but also to the promotion of disease [32]. This may affect the presence of other organisms in the conjunctiva. Indeed, although conflicting evidence exists, studies of human trachoma have reported that chlamydial disease is associated with a decrease in microbial richness and diversity compared to healthy controls [33,34]. Innate immune responses form a key part of the host response to chlamydial infection and, although only speculation, it is possible that upregulation of these processes at the mucosal surface may inadvertently impact on infection by other organisms such as the novel virus detected in this study [32].

These results highlight our limited knowledge of the diversity of novel microorganisms present in unique Australian fauna such as the koala. The clinical impact of Burpengary virus on koala health at the individual and population levels is unclear, and like many other studies of this marsupial is limited by the logistical challenges associated with sampling these animals in the field. The apparently inverse association with chlamydial ocular disease appears to add another dimension to the complex and multi-factorial nature of chlamydial disease in this host [32]. Future studies will be required to characterise the significance of the relationship between this novel virus and the major pathogen of the koala, *C. pecorum*.
